# Manufacture of Three-Dimensional Optofluidic Spot-Size Converters in Fused Silica Using Hybrid Laser Microfabrication

**DOI:** 10.3390/s22239449

**Published:** 2022-12-02

**Authors:** Jianping Yu, Jian Xu, Aodong Zhang, Yunpeng Song, Jia Qi, Qiaonan Dong, Jianfang Chen, Zhaoxiang Liu, Wei Chen, Ya Cheng

**Affiliations:** 1 School of Physics Science and Engineering, Tongji University, Shanghai 200092, China; 2 Shanghai Institute of Optics and Fine Mechanics, Chinese Academy of Sciences, Shanghai 201800, China; 3 XXL—The Extreme Optoelectromechanics Laboratory, School of Physics and Electronic Science, East China Normal University, Shanghai 200241, China; 4 University of Chinese Academy of Sciences, Beijing 100049, China; 5 Engineering Research Center for Nanophotonics and Advanced Instrument, School of Physics and Electronic Science, East China Normal University, Shanghai 200241, China

**Keywords:** femtosecond laser microfabrication, carbon dioxide laser irradiation, glass microchannels, laser-assisted chemical etching, optofluidic waveguides, spot-size converters

## Abstract

We propose a hybrid laser microfabrication approach for the manufacture of three-dimensional (3D) optofluidic spot-size converters in fused silica glass by a combination of femtosecond (fs) laser microfabrication and carbon dioxide laser irradiation. Spatially shaped fs laser-assisted chemical etching was first performed to form 3D hollow microchannels in glass, which were composed of embedded straight channels, tapered channels, and vertical channels connected to the glass surface. Then, carbon dioxide laser-induced thermal reflow was carried out for the internal polishing of the whole microchannels and sealing parts of the vertical channels. Finally, 3D optofluidic spot-size converters (SSC) were formed by filling a liquid-core waveguide solution into laser-polished microchannels. With a fabricated SSC structure, the mode spot size of the optofluidic waveguide was expanded from ~8 μm to ~23 μm with a conversion efficiency of ~84.1%. Further measurement of the waveguide-to-waveguide coupling devices in the glass showed that the total insertion loss of two symmetric SSC structures through two ~50 μm-diameter coupling ports was ~6.73 dB at 1310 nm, which was only about half that of non-SSC structures with diameters of ~9 μm at the same coupling distance. The proposed approach holds great potential for developing novel 3D fluid-based photonic devices for mode conversion, optical manipulation, and lab-on-a-chip sensing.

## 1. Introduction

The manipulation of the mode field of optical waveguides is of vital importance for developing advanced optical transmission, optical interconnection, and optical sensing techniques in integrated optics, photonic microsystems, and lab-on-a-chip devices. In particular, to achieve efficient coupling from optical fibers to on-chip photonic circuits for reducing the mode field mismatching, developing high-performance on-chip waveguide-fiber couplers is of vital importance. Currently, to improve the fiber–chip coupling efficiency, many kinds of on-chip waveguide-fiber couplers, such as grating couplers [1,2], tapered spot-size converters (SSC) [3,4,5,6,7], adiabatically tapered fibers [8,9,10], and vertical couplers [11,12], have been developed. Among these optical couplers, SSC structures feature a smooth transition of the mode field and a relatively simple configuration for on-chip photonic integration. However, such SSC structures usually have planar configurations, because conventional lithographical fabrication methods are employed, which have inherent limitations for achieving the simultaneous modulation of transverse and longitudinal mode fields [6,7,13,14]. To achieve such modulation, the fabrication of three-dimensional (3D) SSC structures is indispensable, but it remains a challenge for most current microfabrication techniques in terms of fabrication procedure and cost-effectiveness [15,16,17,18]. For instance, although 3D tapered SSC structures can be manufactured using a grayscale lithographic method, the whole process is relatively complex and limited to the fabrication of 3D surface microstructures [15]. Meanwhile, for the 3D fabrication of waveguide-based photonic devices in transparent dielectrics, femtosecond (fs) laser inscription based on the modulation of the refractive index around the focal volume has been recognized as a powerful technique in the past two decades. Recently, adiabatic mode-field converters in glass have been successfully demonstrated using direct fs laser writing [19]. However, the flexibility of the refractive index change around the laser-modified region is relatively limited, and the optical properties of the waveguide structures are easily affected by the polarization state of the laser beam, as well as the writing direction. Therefore, developing a new fabrication technique for 3D SSC structures is highly desirable.

Compared with a solid waveguide device, an optofluidic waveguide is a type of device in which a liquid solution serves as the waveguide core encapsulated in a proper cladding layer, such as a microchannel. By modulating the properties of the liquid solution that fills the microchannel, the optical properties of the waveguide can be flexibly changed, enabling a series of functional photonic devices, such as optofluidic waveguides [20,21,22,23], optofluidic Mach–Zehnder interferometers [24], and optofluidic waveguide lasers [25]. However, the fabrication of a 3D optofluidic SSC structure has not yet been explored. To achieve high-efficiency spot-size conversion [13,14], a tapered SSC structure satisfying the adiabatic conversion condition is a simple and convenient choice. In principle, the use of a tapered hollow microchannel could be a promising approach to forming an optofluidic SSC structure. In this work, we demonstrate a facile and reliable approach for the fabrication of 3D optofluidic SSC devices based on the combination of fs laser-assisted chemical etching and carbon dioxide (CO_2_) laser-induced thermal reflow. First, a 3D glass microchannel structure consisting of a tapered channel with a taper angle of 1° and transverse length of 1.3 mm and two straight and uniform channels with different diameters was designed and fabricated by spatially shaped fs laser using slit-beam shaping, followed by selective chemical etching. Second, the internal surfaces of the whole glass channel structures were smoothed by CO_2_ laser irradiation [23]. Finally, liquid-core solutions were introduced into the laser-machined microchannel to form an optofluidic SSC structure. In the fabricated optofluidic SSC device, the input spot size of ~8 μm was expanded to the output spot size of ~23 μm with a ~84.1% conversion efficiency. Furthermore, an on-chip optofluidic device including two symmetric SSC structures with an interval of 250 μm and coupling channel diameter of ~50 μm was fabricated. Additionally, the measured total insertion loss of the device was ~6.73 dB, which was only about half that of the optofluidic device based on straight channel structures with diameters of 9 μm. With the proposed approach, the customized manufacture of various optofluidic SSC structures can be achieved in a facile manner for the flexible manipulation of the mode-field sizes of the waveguides. Moreover, the fabricated SSC structures could offer larger spot sizes and longer coupling distances for developing low-loss and high-performance optofluidic microsystems.

## 2. Experimental

**Figure 1:**
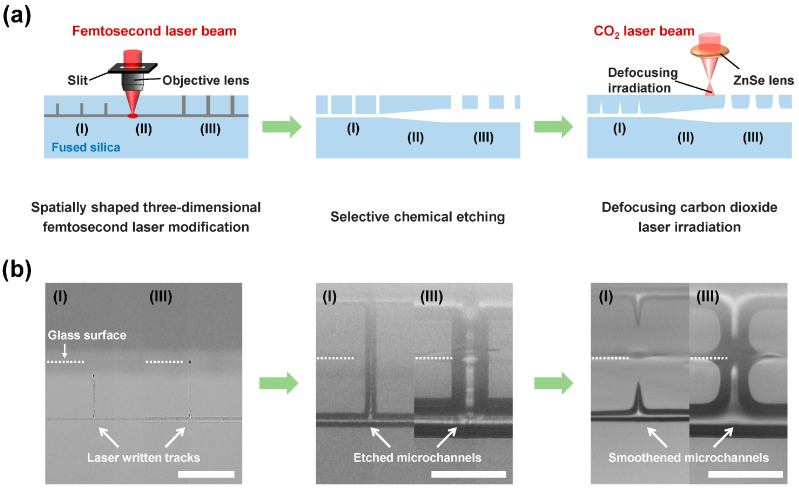
(**a**) Schematic of the fabrication procedure for a spot-size converter channel structure in fused silica glass, which consists of three main steps: spatially shaped three-dimensional fs laser modification (left), selective chemical etching (middle), and defocusing CO_2_ laser irradiation (right). In the left panel, gray lines represent laser-written tracks, which include the left vertical lines beneath the glass’s surface with a depth of ~18 µm (region I), a horizontal line embedded in glass with a depth of ~100 µm (region II), and the right vertical lines directly exposed on the glass surface (region III). In the middle panel, the sizes of the etched channel and the extra-access ports on the left (region I) are smaller than those on the right (region III), as the preferential etching of the laser-written tracks on the right occurs, which leads to the formation of the tapered channel structure in the middle (region II). In the right panel, the internal surface of the whole channel is smoothed by CO_2_ laser irradiation. (**b**) Side-view optical micrographs of the corresponding glass microstructures at each step in (**a**). White dashed lines and scale bars in (**b**) represent the glass surface and 100 µm, respectively.

In most experiments, fused silica (JGS1) samples with sizes of 20 mm × 10 mm × 1 mm were used as the processing glass substrates. Figure 1a illustrates a schematic of the fabrication procedure of a spot-size converter channel structure in glass. First, spatially shaped fs laser direct writing based on slit beam shaping was used to create modified micropatterns in fused silica. As shown in the left panels of Figure 1a,b, the micropatterns included a string of vertical lines beneath the glass surface, with a depth of ~18 µm (see region I), a horizontal line embedded in glass, with a depth of ~100 µm (see region II), and a string of vertical lines directly exposed on the glass surface (see region III). The spacing of the vertical lines in regions I and III and the length of the horizontal line in region II were set to 300 μm and 1.3 mm, respectively. To achieve a tapered channel structure in region II, the vertical lines and the horizontal line in region III were first removed and the embedded vertical lines in region I started to be removed until the glass matrix above those lines was etched away. An ultrashort laser system (Light Conversion, Pharos 20 W) with a central wavelength of 1030 nm, a pulse duration of 270 fs, and a repetition rate of 200 kHz was employed for 3D laser modification. For beam shaping, a 600 μm-wide slit was placed before an objective lens with a numerical aperture of 0.80 to ensure a spatially symmetric modification around the focal volume, and the orientation of the slit was along the laser writing direction, which was perpendicular to the laser polarization direction. For direct laser writing, the glass sample was mounted on a 3D motorized positioning stage (Coretech, CFT-200XY-TTL005) with a translation speed of 1 mm/s. After passing through the slit, the measured pulse energy before the objective lens was 1.5 μJ. Second, the laser-irradiated glass sample was immersed in a 10 M KOH solution at 90 °C in an ultrasonic bath for 12 h to remove the laser-modified regions. After chemical etching, a microchannel structure including a ~15 μm-diameter channel, a ~1.3 mm-long tapered channel, a ~58 μm-diameter channel, and a string of extra-access ports distributed along the channel with a spacing of 300 μm was fabricated in glass (see the middle panels of Figure 1a,b). The introduction of a string of extra-access ports aimed to promote the homogeneity of the straight channel around the tapered channel. Finally, the etched channel sample was smoothed by defocusing the CO_2_ laser irradiation. Direct CO_2_ laser beam writing on the glass surface was performed along the trajectory of the embedded microchannel using a CO_2_ laser source (Synard, Ti100-HS) and a ZnSe lens with a focal length of 15.9 cm in a defocused manner [23,26,27]. The average power of the CO_2_ laser beam, the writing speed, and the defocusing distance were set at 16.5 W, 1.6 mm/s, and 5 cm, respectively. After CO_2_ laser irradiation, the sizes of all extra-access ports exhibited significate shrinkage; in particular, the ports with smaller sizes on the left could be sealed as shown in the right panel of Figure 1b. To form an optofluidic waveguide, a fresh mixture of n-decane and liquid paraffin with a ratio of 1:3 was prepared with an estimated refractive index of 1.4521 and subsequently filled the smoothed channel [23].

## 3. Results and Discussion

### 3.1. Fabrication of 3D Tapered Channel Structures

**Figure 2:**
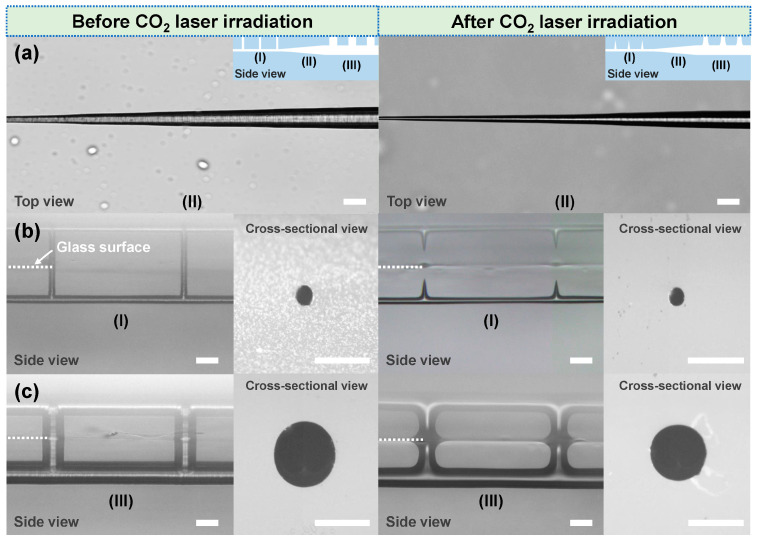
Optical microscopy images of different regions of 3D laser-machined glass microchannel structures before and after CO_2_ laser irradiation. (**a**) Top-view optical micrographs of the tapered microchannel structure (region II). Each inset in (**a**) represents a side-view schematic of the whole microchannel structure, which consists of three regions (I, II, and III). Side-view optical micrographs of (**b**) region I and (**c**) region III of the microchannel structure. Each inset in (**b**,**c**) shows a cross-sectional view of the microchannel in the corresponding region. The white dashed lines in (**b**,**c**) indicate the glass surface. The scale bar in each figure represents 50 µm.

Figure 2a shows top-view optical micrographs of the etched tapered microchannel structure (region II) before and after CO_2_ laser irradiation. Before CO_2_ laser irradiation, the diameters of the left and right ends of the tapered channel in region II were about 15 μm and 58 μm, respectively, which were the same as the diameters of two straight channels in region I and region III. The formation of a tapered microchannel structure was mainly due to the spatially asymmetric etching based on the etching selectivity. To create etching asymmetry, the vertical lines and the horizontal line in region III connected to the glass surface were preferably etched to form a hollow microchannel in region III, and the etching of the horizontal line in region II was then triggered from the right side. With the increase in the etching time, the gradient etching of the horizontal line in region II was carried out before the glass matrix above the vertical lines in region I was etched away. Finally, after etching the straight channel in region I, a 1.3 mm-long tapered channel structure with a taper angle of 1° was obtained. After CO_2_ laser irradiation, the size of the whole tapered microchannel structure exhibited uniform shrinkage with a nearly unchanged taper angle (~1°). As shown in the right panel of Figure 2a, the diameters of the left and right ends of the tapered channel decreased to 9 μm and 50 μm, respectively. Moreover, the inner surfaces of the whole channel structures were smoothed by direct CO_2_ laser writing, which can be identified from the right panels of Figure 2a–c. As the fused silica had strong absorption of the laser energy at a wavelength of ~10.6 μm, the temperature of the laser-irradiated area rapidly increased under the CO_2_ laser heating, and then the molten pool generated in situ on the glass surface combined with the thermal diffusion penetrated the glass matrix. Regarding the depth of the etched whole microchannel structure (less than 100 μm), which was in the range of the effective thermal diffusion distance (several hundreds of micrometers), the melting of the internal surface of the channel occurred due to the thermal reflow effect and then resolidified to form a newly polished surface with improved smoothness [23]. For the extra-access ports, following CO_2_ laser irradiation, they exhibited different shape changes due to the difference in the diameters in regions I and III. The extra-access ports in region I were fully sealed during CO_2_ laser direct writing, as previously reported [26,27], while the ports in region III were narrowed due to their larger diameters. A possible reason is that the heat-affected zone generated by the defocusing CO_2_ laser irradiation could not provide enough melted glass to fill the ports with 50 μm diameters and 50 μm lengths. Further optimization of the heat-affected zone and the spatial sizes of the ports will be explored in the future. Regarding the cross-section control of the microchannels, the etched straight microchannels with nearly symmetric cross-sections in regions I and III could be obtained using an optimized slit beam shaping scheme (see the insets of the left panels of Figure 2b,c), which was consistent with the previous results [23]. After CO_2_ laser irradiation, the circular-shaped cross-sections could remain unchanged, although the diameters of the channels uniformly decreased due to the surface tension effect.

### 3.2. Characterization of Optofluidic SSC Structures

**Figure 3:**
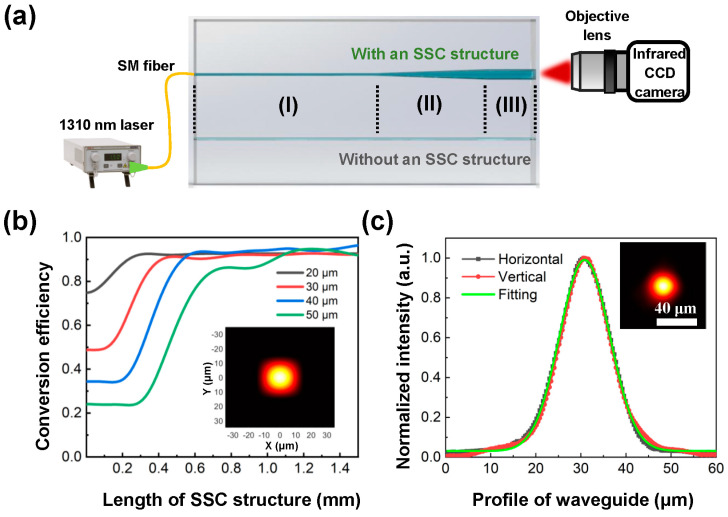
(**a**) Schematic of the optical layout for the characterization of optofluidic waveguide devices with and without an SSC structure in glass. (**b**) Numerically simulated mode-field conversion efficiency of the SSC structure under different exit port lengths and diameters. The inset in (**b**) shows a simulated output mode field at an exit port of a 50 μm-diameter channel. (**c**) Measured output mode field at an exit port of a 50 μm-diameter channel of the SSC structure.

After fabricating the 3D tapered channel structure in fused silica, the channel was filled with the mixture of n-decane and liquid paraffin as a liquid-core solution to form an optofluidic SSC structure. Figure 3a illustrates a schematic of the optical layout for the mode-field characterization of optofluidic waveguide devices with and without a tapered SSC structure. The spacing between the extra-access ports in the fabricated devices was 300 μm. To evaluate the mode-field conversion efficiency of the optofluidic SSC structure, a tapered channel structure and a straight channel with the same length were simultaneously fabricated, as shown in Figure 3a. To avoid the influence of the unsealed extra-access ports on the measurement of the conversion efficiency, the length of the 50 μm-diameter straight channel in region III was set at 100 μm to ensure the etching homogeneity without the extra-access ports. After the introduction of the liquid-core solution to the whole channel, a 1310 nm laser was coupled to the straight ~9 μm-diameter microchannel in region I through a single-mode fiber; then, the output mode field of the straight 50 μm-diameter channel in region III was captured by an InGaAs CCD camera (Hamamatsu Photonics K.K., Hamamatsu City, Japan, C12741-03) through the projection of an objective lens. To reduce the coupling loss, the liquid-core solution filled the gap between the fiber tip and the end face of the glass microchannel as a refractive-index-matching solution. Figure 3b shows the simulated conversion efficiencies of the fundamental mode field of the tapered SSC structures with different lengths and different output diameters. One can see that, to improve the mode-field conversion efficiency, the larger the output diameter, the longer the required length of the tapered structure. For a tapered SSC structure with a diameter of 50 μm, the maximum mode-field conversion efficiency of ~95% could be theoretically obtained when its length was greater than 1.2 mm. Moreover, when the length of the tapered structure was set at 1.3 mm, the variation in the output diameter had little effect on the simulated conversion efficiency, as shown in Figure 3b. In addition, according to the simulation, a single-mode ~25 μm diameter waveguide output could be obtained through a tapered SSC structure with an output diameter of 50 μm (see the inset of Figure 3b). Therefore, the length of the tapered SSC structure for processing was set at 1.3 mm for an output diameter of 50 μm and the conversion efficiency of the processed tapered SSC structure was experimentally tested. As previously demonstrated, a ~9 μm-diameter circular microchannel facilitated a single-mode optofluidic waveguide propagation with a mode-field diameter of about 8 μm [23]. The measured results showed that a ~23 μm-diameter single-mode waveguide output could be obtained through the fabricated optofluidic SSC structure, in which both the horizontal and longitudinal profiles were close to a Gaussian distribution, as shown in Figure 3c. Therefore, through an optofluidic tapered SSC structure with a length of 1.3 mm, a fundamental mode with a diameter of about 8 μm was successfully converted into a fundamental mode with a diameter of ~23 μm, which is close to the simulation result (~25 μm), as shown in the inset of Figure 3b. However, although the actual length of the fabricated tapered channel was 1.3 mm, the mode-field conversion efficiency of the tapered SSC structure was 84.1% from the experimental measurement, which was lower than the simulation value. A possible reason was the losses caused by the residual roughness of the inner walls of the tapered channel after defocusing CO_2_ laser treatment.

### 3.3. Coupling Loss Evaluation of SSC Devices

**Figure 4:**
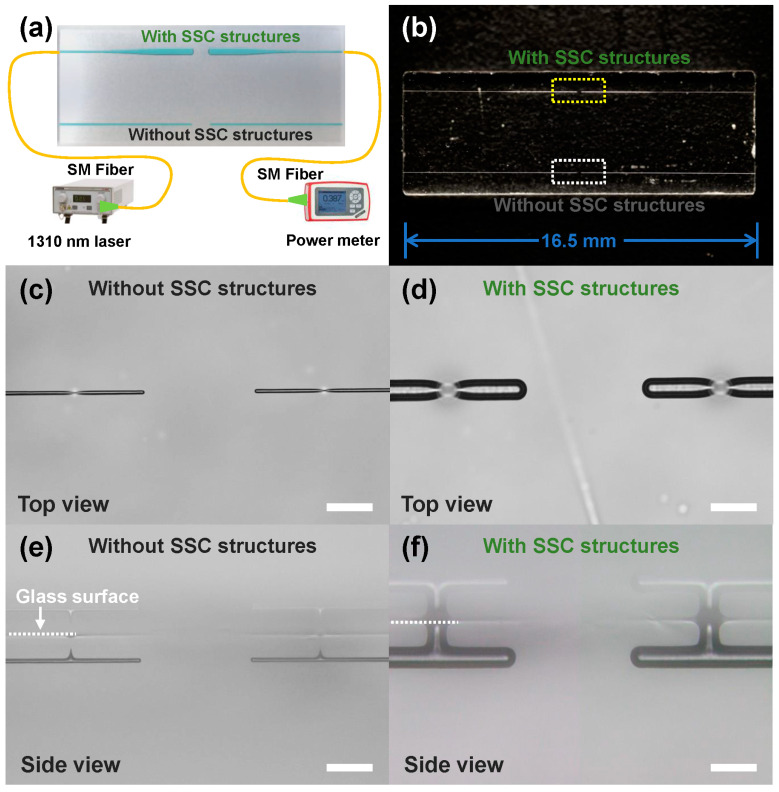
(**a**) Schematic of the experimental setup for the coupling loss measurement of optofluidic devices with and without SSC structures. (**b**) Photograph of a glass microchannel sample including symmetric SSC and non-SSC channel structures with an interval of 250 µm. (**c**) Top-view and (**e**) side-view optical micrographs of the coupling terminals of two ~9 µm-diameter non-SSC structures, which are indicated by a dashed white rectangle in (**b**). (**d**) Top-view and (**f**) side-view optical micrographs of the coupling terminals of two ~50 µm-diameter SSC structures, which are indicated by a dashed yellow rectangle in (**b**). White dashed lines in (**e**,**f**) indicate the glass surface. The scale bars in (**c**–**f**) represent 100 μm.

To demonstrate the capacity of SSC structures for low-loss on-chip coupling, two symmetric SSC channel structures and two symmetric non-SSC channel structures with the same length were simultaneously manufactured inside glass, and their insertion losses were individually measured as demonstrated in Figure 4a,b. For symmetric SSC and non-SSC channel structures with the same total length of 16.5 mm, ~50 μm- and ~9 μm-diameter outputs were coupled to each other with an interval of 250 μm, respectively, as shown in Figure 4c,d. The measured insertion loss of the symmetric SSC structures was ~6.73 dB, which was only about half that of non-SSC structures (~13.56 dB), as the SSC structures provided a larger mode size and a longer Rayleigh distance for efficient coupling. The calculation formula of the Rayleigh distance (*Z_R_*) is as follows:

(1) $$z_{R}=\frac{\pi \omega_{0}^{2}}{\lambda }\;$$

In the equation, for the fabricated SSC waveguide structure, the diameter of the output mode field was ~23 μm and the wavelength of the laser was 1310 nm, and the calculated Rayleigh distance was ~317 μm, which was larger than the coupling distance of 250 μm. In contrast, for the ~8 μm-diameter mode field of the non-SSC optofluidic waveguide, the corresponding Rayleigh distance was only ~48 μm. Therefore, at the same coupling distance of 250 μm, the optofluidic SSC structure provided superior performance for low-loss coupling. Moreover, it can be seen from the side-view optical micrographs in Figure 4e,f that the coupling terminals of the microchannels exhibited hemispherical shapes due to the nature of the glass etching. For the SSC structure, the ~50 μm diameter hemispherical structures acted as quasi-microlens during waveguide coupling. The estimated focal length of this quasi-microlens was about 6.8 mm, which was much larger than the coupling distance (250 μm), leading to a reduction in the divergence angle of the optofluidic waveguide and an improvement in coupling efficiency.

## 4. Conclusions

The facile fabrication of tapered microchannels in glass substrates based on the nature of fs laser-assisted etching was demonstrated. With the further combination of CO_2_ laser irradiation and the introduction of the liquid-core solution, optofluidic waveguide devices were created for mode-field conversion. By using the fabricated optofluidic SSC structure, an input spot size of ~8 μm was expanded to an output spot size of ~23 μm with a ~84.1% conversion efficiency. Furthermore, the insertion loss measurement of the optofluidic waveguide–waveguide coupling structures showed that the utilization of SSC structures through the 50 μm-diameter coupling ports provided a higher coupling efficiency at the same coupling distance compared with the case of ~9 µm-diameter non-SSC structures. With the proposed technique based on the nature of laser-induced etching selectivity, the tunability of the mode-field diameter with a wide range can be achieved and it can be tailored flexibly. Moreover, regarding its superior features for mode-field conversion and coupling efficiency, we believe that the manufactured optofluidic SSC structure will hold great potential for on-chip detection with high sensitivity in the fields of advanced photonics, lab-on-a-chip devices, biochips, etc.

## Data Availability

The data presented in this study are available on request from the corresponding authors.
